# Multiplex Partial Nephrectomy, Repeat Partial Nephrectomy, and Salvage Partial Nephrectomy Remain the Primary Treatment in Multifocal and Hereditary Kidney Cancer

**DOI:** 10.3389/fonc.2017.00244

**Published:** 2017-10-20

**Authors:** Joseph A. Baiocco, Adam R. Metwalli

**Affiliations:** ^1^Urologic Oncology Branch, National Institutes of Health (NIH), Bethesda, MD, United States

**Keywords:** multiplex partial nephrectomy, salvage partial nephrectomy, repeat partial nephrectomy, multifocal kidney cancer, hereditary kidney cancer, renal transplant, hereditary cancer syndromes

## Abstract

The standard of care treatment for solitary renal cell carcinoma (RCC) tumors 4 cm or less is partial nephrectomy (PNx). However, multifocal kidney cancer presents unique challenges for treating physicians. Historically, total nephrectomy and hemodialysis with possible renal transplant later was the primary therapeutic strategy for these patients. Later, as nephron sparing surgical approaches improved, PNx became the standard of care for patients presenting with multifocal and hereditary RCC. Surgeries to remove multiple renal tumors simultaneously produce different perioperative outcomes and increased risk of complications. Due to these differences in technique and outcomes, the term multiplex partial nephrectomy (MxPNx) has been coined to designate these differences. Here, we discuss the role that MxPNx continues to play in multifocal RCC.

The standard of care treatment for solitary renal cell carcinoma (RCC) tumors 4 cm or less is partial nephrectomy (PNx) (1). However, multifocal RCC creates distinct difficulties for providers. More than 30 years ago, radical nephrectomy (RNx) and renal replacement therapy (RRT) such as hemodialysis or peritoneal dialysis were considered the principal treatment in this patient population. But as surgical techniques evolved and a greater understanding of the biology of these tumors was attained, PNx became the standard of care for these patients as oncologic efficacy proved to be equivalent to RNx, and quality of life (QOL) metrics for patients with native renal function far surpassed QOL for those on dialysis. Because procedures removing multiple renal tumors as a single surgery require unique and advanced surgical techniques, a significantly higher rate of intraoperative and postoperative complications risks are expected (2–4). The term multiplex partial nephrectomy (MxPNx) was conceived to emphasize the fact that perioperative outcomes for PNx with a solitary tumor are markedly different than those for PNx for multiple tumors (2, 5).

When choosing an initial treatment modality, providers and patients must also recognize that in multifocal and hereditary RCC, tumors may recur after PNx or MxPNx. Consequently, repeat renal surgery (RRS) and salvage renal surgery (SRS) may be necessary and have been shown to be exceedingly effective in preserving renal function despite being highly challenging and morbid procedures (6–9). The subset of multifocal and hereditary RCC patients who do require RRS and SRS tend to undergo these procedures at average intervals greater than 5 years while enduring complication rates greater than 50% (10). However, the overall benefit of these difficult surgeries has been demonstrated not only with an exceedingly low rate of RRT (3.9–23%) and, in fact, minimal decreases in overall renal function (7–10), but also in terms of economic burden to the health care system despite the added expense due to complications (11).

Yet in this era of increased emphasis on cost and outcomes, the high complication rate of these procedures has prompted a reconsideration of this approach. As immunosuppressive medications have improved, so too have results for renal allograft survival (12). Evidence supporting improved outcomes in renal transplant over the past two decades has steadily grown. Which now raises the question whether nephrectomy and transplant should be revisited as a viable strategy for patients with multifocal and hereditary RCC. A recent analysis of outcomes demonstrated 5-year living donor graft survival rates improved from less than 30% in 1991 to over 60% in 2008. When adjusted for patient survival, the 5-year graft survival rate was nearly 90% in 2008 (13). Furthermore, other reports suggest that renal allograft half-life may be as long as 16 years in some patient populations (14). These results suggest that the ongoing improvements in immunosuppression and allograft survival may ultimately make this approach a more appealing strategy for patients with multifocal and hereditary RCC.

However, an important aspect of these data to consider is that the preponderance of patients in these studies required transplantation due to medical comorbidities like hypertension, diabetes mellitus, and hyperlipidemia resulting in end-stage renal disease (ESRD). But the chronic kidney disease (CKD) and ESRD that may develop in some patients with multifocal and hereditary kidney cancer has a distinct natural history because it is usually a result of the multiple surgeries. This renal dysfunction tends to be more stable rather than progressive as seen in CKD secondary to medical comorbidities (15, 16). Moreover, patients with hereditary and multifocal RCC who progress to dialysis are generally younger and with better overall health than patients on dialysis due to medical ESRD (17). As a result, the overall life expectancy for patients with ESRD and a history of multifocal or hereditary kidney cancer may be longer than would be seen in a patient population with more medical comorbidities. Thus, even with tremendous improvements in allograft survivals, a 16-year allograft half-life may not be adequate for the life expectancy of this patient population. Further research to address this issue is needed.

Over essentially the same period that transplant outcomes were markedly improving, healthcare systems and insurers have begun placing greater emphasis on treatment outcomes and expenses in an effort to minimize complications and curb the skyrocketing costs of healthcare. In this context, the economic burden of MxPNx, RRS, and SRS for multifocal RCC should be reassessed (18). Nassir et al. determined the mean cost of RNx and transplant to be just above $65,000 using US Medicare claims (19). In contrast, other analyses using Medicare reimbursement rates, showed that the cost of uncomplicated completion nephrectomy and 5 years of dialysis was less economically favorable compared to RRS despite deliberate underestimation of dialysis costs at only $40,000–$50,000 annually. Cost analysis modeling demonstrated the comparative cost savings of SRS in contrast to the estimated costs of a hypothetical total nephrectomy and dialysis cohort in less than 9 months. To account for the high complication rates of SRS, a second model was created deliberately overestimating cost of SRS was analyzed, and the benefit was still achieved in under 1 year (11). Juxtaposing these studies indicates that MxPNx, RRS, and SRS are cost-effective when compared to completion RNx and dialysis. And if these data are not convincing enough, the most compelling reason to maintain MxPNx, RRS, and SRS as the standard of care for multifocal and hereditary RCC is the chronic shortage of donor organs.

The shortage of available donor kidneys has been well documented for more than four decades (20, 21). To alleviate this deficit and shorten waiting times, so-called expanded pools and donor exchanges were created with little success in alleviating the problem. However, even with the creation and use of expanded donor criteria and kidney exchange programs, median wait times continue to increase. For example, in 2009, the median wait time for any kidney was 4.5 years, whereas in 2003 the median wait time was only 3 years (13, 22). The increased wait time is a result of higher demand due to a growing number of patients on the kidney waiting list with a stagnant rate of organ donation. From 2003 to 2013, the number of patients on the kidney transplant waiting list doubled. This dramatic change in demand is reflective of the fact that diabetic nephropathy, which historically was the most common cause of ESRD, is now also is the fastest rising cause of ESRD. This diabetes epidemic is also decreasing donor kidney availability, as kidneys are increasingly being rejected because the donor has or had diabetes (13, 23). Thus—despite the high rate of peri- and postoperative complications and marked improvements in immunosuppression and transplant graft and patient survival—RRS and SRS continue to be the optimal treatment for multifocal and hereditary kidney cancer.

In summary, as outcomes and inpatient re-admissions continue to gain administrative importance, initial MPNx, RRS, and SRS may be perceived as increasingly unappealing treatment options by physicians and patients with multifocal and hereditary RCC alike. In addition, the improving survival and decreasing costs of renal transplant potentially provide a reasonable alternative to this strategy and a rational argument can be made for making nephrectomy and transplant the primary treatment option for these patients. However, widespread implementation of completion RNx and transplant is not feasible at this time due to the profoundly worsening shortage of donor kidneys. Due to the unchecked epidemic of obesity and diabetes in the United States, no short-term prospects for increased donor organs is on the horizon. Combine that with the added cost of RNx and transplant compared to RRS and SRS, and the case for RNx and transplant as the preferred approach weakens considerably. Thus, despite the morbidity and manifold challenges associated with MPNx, RRS, and SRS; these complex surgeries continue to be the optimal primary treatment of hereditary and multifocal RCC.

## Author Contributions

Article concept and design; drafting of the article; critical revision of the article: JB and AM.

## Conflict of Interest Statement

The authors declare that the research was conducted in the absence of any commercial or financial relationships that could be construed as a potential conflict of interest.
